# Calculating the peak skin dose resulting from fluoroscopically guided interventions. Part I: Methods

**DOI:** 10.1120/jacmp.v12i4.3670

**Published:** 2011-11-15

**Authors:** A. Kyle Jones, Alexander S. Pasciak

**Affiliations:** ^1^ Department of Imaging Physics, Division of Diagnostic Imaging The University of Texas M. D. Anderson Cancer Center Houston TX 77030; ^2^ Department of Radiology University of Tennessee Medical Center Knoxville TN 37920

**Keywords:** peak skin dose, fluoroscopy, dose report, radiation injury

## Abstract

While direct measurement of the peak skin dose resulting from a fluoroscopically‐guided procedure is possible, the decision must be made a priori at additional cost and time. It is most often the case that the need for accurate knowledge of the peak skin dose is realized only after a procedure has been completed, or after a suspected reaction has been discovered. Part I of this review article discusses methods for calculating the peak skin dose across a range of clinical scenarios. In some cases, a wealth of data are available, while in other cases few data are available and additional data must be measured in order to estimate the peak skin dose. Data may be gathered from a dose report, the DICOM headers of images, or from staff and physician interviews. After data are gathered, specific steps must be followed to convert dose metrics, such as the reference point air kerma $\left(K_{a,r}\right)$ or the kerma area product (KAP), into peak skin dose. These steps require knowledge of other related factors, such as the f‐factor and the backscatter factor, tables of which are provided in this manuscript. Sources of error and the impact of these errors on the accuracy of the final estimate of the peak skin dose are discussed.

PACS numbers: 87.59.Dj, 87.53.Bn

## I. INTRODUCTION

The PSD (see Table 1 for definitions of this and other abbreviations used in this article) is the most useful quantity for estimating the risk of a deterministic skin injury from an FGI. In practice, the PSD is very difficult to measure,^(^ ^1^ ^)^ and the decision to measure the PSD must be made a priori. Other quantities related to the PSD, such as $K_{a,r}$ or KAP, are often used as surrogates for the PSD. However, as the PSD can be substantially different from $K_{a,r}$,^(^ ^2^ ^)^ it is necessary to estimate the PSD using available data when making decisions regarding the medical management of a patient believed to have received a substantial skin dose.

**Table 1:** Abbreviations used in this manuscript.

*Term*	*Abbreviation*	*Definition*
Peak skin dose	PSD	The highest dose to a single area of the skin, units of Gy.
Fluoroscopically‐guided intervention	FGI	Any medical intervention using fluoroscopy for guidance.
Reference point air kerma	$K_{a,r}$	Cumulative air kerma at the interventional reference point, units of Gy.
Kerma area product	KAP	Product of air kerma and X‐ray field size, units of Gy‐cm^2^.
Digital Imaging and Communications in Medicine	DICOM	Standard for communication used by medical imaging equipment.
Interventional reference point	IRP	Point located 15 cm back towards the focal spot from the isocenter of a C‐arm fluoroscope^(^ ^3^ ^)^.
Source‐to‐image distance	SID	Distance from the focal spot to the center of the image receptor, units of mm.
Field of view	FOV	Size of the X‐ray field at the image receptor, units of cm.
Entrance skin air kerma	ESAK	Air kerma measured at the entrance surface of the patient, units of Gy.
Source‐to‐patient distance	SPD	Distance from the focal spot to the entrance surface of the patient, units of mm.
Backscatter factor	BSF	Factor that is applied to calculate entrance surface dose from the ESAK, accounts for the fact that many X‐rays of diagnostic energy are backscattered in tissue, unit‐less.
f‐factor	f	Factor used to convert exposure to dose in tissue, units of Gy/R. A similar, unit‐less quantity can be used to convert from air kerma to dose in tissue.
Digital acquisition series	DAS	A series of images generated using digital acquisition imaging.
Fluoroscopic reference point air kerma	$K_{a,r}(f)$	Total reference point air kerma resulting from fluoroscopic imaging, units of Gy.
Digital acquisition reference point air kerma	$K_{a,r}(d)$	Total reference point air kerma resulting from digital acquisition imaging, units of Gy.
Projected X‐ray field size on the skin	$A_{\text{skin}}$	The area of the projected X‐ray field at the entrance surface of the patient, units of cm^2^.

In the past, estimates of PSD were necessarily crude, as little information directly related to the procedure was available. With the advent of published standards from the International Electrotechnical Commission (IEC)^(^ ^3^ ^)^ and a subsequent change to United States law,^(^ ^4^ ^)^ terminology was standardized and reporting of $K_{a,r}$ was required. In addition, the DICOM standard made available more information directly related to FGI, including geometrical and technical factors. By taking full advantage of the availability and standardization of this information, more accurate estimates of PSD can be made and patients can be managed more appropriately.

This review describes methods that can be used to estimate the PSD resulting from an FGI across a range of clinical scenarios, with different sources of information available in each scenario. This manuscript does not address the direct measurement of PSD, as this has been addressed previously by other authors.^(^ ^1^ ^)^

## II. ESTIMATING THE CONTRIBUTION OF DIGITAL ACQUISITION IMAGING TO THE PEAK SKIN DOSE

### A. Determination of the reference point air kerma

The first step in estimating the PSD delivered during a procedure is to determine $K_{a,r}$. This task can be quite simple or quite complex, depending on the data available and whether or not these data are being collected. The IEC has long had a standard requiring the display of the $K_{a,r}$,^(^ ^3^ ^)^ whereas the United States Food and Drug Administration (FDA) requires that any fluoroscope manufactured after June 2006 display the cumulative air kerma^(^ ^4^ ^)^ at either the IRP^(^ ^3^ ^)^ or a different point that is “deemed by the manufacturer to represent the intersection of the x‐ray beam entrance surface and the patient skin.”

**Scenario 1: Reference point air kerma available**

Retrieving or calculating the $K_{a,r}$ when using a fluoroscope that complies with the IEC standard or is subject to the FDA requirement is relatively simple. $K_{a,r}$ is available directly if the fluoroscope complies with IEC standards, and it can be calculated using an inverse square law correction if the manufacturer's reference point is different from the IRP. However, if the $K_{a,r}$ is not explicitly displayed or available, the problem becomes more complex. We consider the following additional scenarios.

**Scenario 2: No dose metrics available, images available for review**

In the absence of machine‐generated dose metrics, the medical physicist must make measurements and use these data, along with a review of the images, to estimate the $K_{a,r}$. In this scenario, it is important to estimate the patient thickness along the direction of the X‐ray beam and to determine the mode of operation of the equipment. The former can be estimated using cross‐sectional images or by reviewing the medical record; the latter is most easily determined by interviewing the staff, including the physician and technologist(s) directly involved with the FGI.

A suitable attenuator (e.g., acrylic or water) can be used to model the patient, and measurements of the digital acquisition air kerma per frame can be performed, in the absence of backscatter, for the mode(s) of operation used during the procedure. $K_{a,r}(d)$ can be estimated by multiplying the measured air kerma per frame by the number of frames.

If the images resulting from the FGI are on a permanent archive but not available locally, one must always consider the possibility that additional images were generated but not archived. It is also important to use the exact modes used during the procedure if possible, as adjunct modes such as DSA involve additional radiation dose from mask images along with a higher input detector air kerma rate than digital acquisition imaging.

**Scenario 3: No dose metrics available, images not available for review**

In this case, it is impossible to make an accurate estimate of the $K_{a,r}$. The RAD‐IR study published data on dose metrics, including $K_{a,r}$, for a variety of FGI.^(^ ^2^ ^)^ These data can be consulted as a starting point, but $K_{a,r}$ will vary greatly with patient size, mode of operation, the skill of the physician, and other factors. If a high PSD is suspected for any reason, the patient should be counseled and carefully monitored for the development of symptoms of a radiation‐induced skin injury. Fluoroscopy time alone should not be used to assess the potential for skin injury, as it is poorly correlated with PSD.^(^ ^1^ ^)^

**Scenario 4: Kerma area product available, but not reference point air kerma**

The $K_{a,r}$ can be estimated from the KAP by removing the dependence on X‐ray field size. KAP is invariant along the focal spot to image receptor axis and, if the X‐ray field size can be determined at any point along this axis, the $K_{a,r}$ can be calculated using known distances. One method for determining the X‐ray field size is by using data from the DICOM header (Table 2). The collimator positions and imager pixel size, along with the SPD and SID, can be used to estimate $A_{\text{skin}}$:

(1) $$A_{skin}=\left[\left(\left|C_{Left}-C_{Right}\right|\times \left(p/10\right)\right)\times \left(\left|C_{Upper}-C_{Lower}\right|\times \left(p/10\right)\right)\right]\times \left(\frac{SPD}{SID}\right)$$

where $C_{x}$ represents the respective collimator position in pixels for a square collimator and *p* is the imager pixel size in mm. $A_{\text{skin}}$ can be estimated similarly for circular or polygonal collimators. $A_{\text{skin}}$ can then be used to estimate $K_{a,r}(d)$:

(2) $$K_{a,r}(d)=\frac{KAP}{A_{skin}}$$

**Table 2:** Data that can be mined from the DICOM header. Data available will vary by manufacturer and model.

*Tag Name*	*Tag Number*	*Location*	*Definition*
kVp	(0018,0060)	X‐RAY ACQUISITION MODULE, X‐RAY ACQUISITION DOSE MODULE, XA/XRF ACQUISITION MODULE	Peak kilovoltage output of X‐ray generator.
Intensifier size	(0018,1162)	X‐RAY ACQUISITION, X‐RAY IMAGE INTENSIFIER MODULE	Diameter of X‐ray intensifier in mm.
Field of view dimension(s)	(0018,1149)	X‐RAY ACQUISITION	Dimensions of image intensifier field of view in mm. If rectangle, row followed by column; if round, diameter.
Imager pixel spacing	(0018,1164)	X‐RAY ACQUISITION	Physical distance between pixels measured at front plane of image receptor housing: row spacing(delimiter)column spacing.
Collimator shape	(0018,1700)	X‐RAY COLLIMATOR MODULE	Shape of collimator: RECTANGULAR, CIRCULAR, POLYGONAL
Collimator left vertical edge	(0018,1702)	X‐RAY COLLIMATOR DIMENSIONS MACRO	Required if (0018,1700)=RECTANGULAR. Location of left edge of collimator with respect to pixels in image (column).
Collimator right vertical edge	(0018,1704)	X‐RAY COLLIMATOR DIMENSIONS MACRO	Required if (0018,1700)=RECTANGULAR. Location of right edge of collimator with respect to pixels in image (column).
Collimator upper horizontal edge	(0018,1706)	X‐RAY COLLIMATOR DIMENSIONS MACRO	Required if (0018,1700)=RECTANGULAR. Location of upper edge of collimator with respect to pixels in image (row).
Collimator lower horizontal edge	(0018,1708)	X‐RAY COLLIMATOR DIMENSIONS MACRO	Required if (0018,1700)=RECTANGULAR. Location of lower edge of collimator with respect to pixels in image (row).
Center of circular collimator	(0018,1710)	X‐RAY COLLIMATOR DIMENSIONS MACRO	Required if (0018,1700)=CIRCULAR. Center of collimator in pixels given as row and column.
Radius of circular collimator	(0018,1712)	X‐RAY COLLIMATOR DIMENSIONS MACRO	Required if (0018,1700)=CIRCULAR. Radius of collimator in pixels along the row direction.
Vertices of polygonal collimator	(0018,1720)	X‐RAY COLLIMATOR DIMENSIONS MACRO	Required if (0018,1700)=POLYGONAL. Multiple values, first set of two are row and column of origin vertex. Two or more pairs of values follow and are the row and column coordinates of other vertices, in pixels.
Distance Source to Patient	(0018,1111)	XA POSITIONER MODULE, XRF POSITIONER MODULE, X‐RAY ACQUISITION DOSE MODULE	Distance in mm from source to isocenter. NOTE: May be distance from source to table for some manufacturers.
Distance Source to Detector	(0018,1110)	XA POSITIONER MODULE, XRF POSITIONER MODULE, X‐RAY ACQUISITION DOSE MODULE	Distance in mm from source to detector center.
Estimated Radiographic Magnification Factor	(0018,1114)	XA POSITIONER MODULE and XRF POSITIONER MODULE	Ratio of Source Image Receptor Distance (SID) over Source Object Distance (SOD)=(0018,1110)/(0018,1111).
Positioner Primary Angle	(0018,1510)	XA POSITIONER MODULE	Position of the X‐ray Image Intensifier about the patient from the RAO to LAO direction where movement from RAO to vertical is positive.
Positioner Secondary Angle	(0018,1511)	XA POSITIONER MODULE	Position of the X‐ray Image Intensifier about the patient from the CAU to CRA direction where movement from CAU to vertical is positive.
Image and Fluoroscopy Area Dose Product	(0018,115E)	X‐RAY ACQUISITION DOSE MODULE	Dose area product in dGy*cm*cm.
Acquired Image Area Dose Product	(0018,9473)	XA/XRF ACQUISITION MODULE	Dose area product to which the patient was exposed in dGy*cm*cm.
Number of frames	(0028,0008)	MULTI‐FRAME MODULE ATTRIBUTES	Number of frames in a multi‐frame image.

* Other information may be available in Modality Performed Procedure Step, including the Radiation Dose Module

If none of this information is available and images are not available for review, the full FOV can be assumed to represent the field size, although this may result in underestimation of the $K_{a,r}$. The FOV can be determined by reviewing images and interviewing the staff.

#### A.1 Mining the DICOM header for data

**Figure 1:**
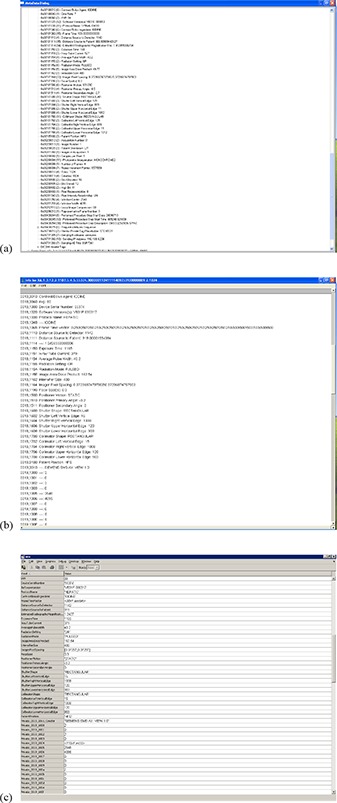
Methods for accessing data in the DICOM header: (a) Section of DICOM header as displayed using PACS, (b) section of DICOM header as displayed using ImageJ, (c) section of DICOM header as displayed using MATLAB.

KAP is a public tag in both the RF and XA DICOM Service Class objects,^(^ ^5^ ^)^ and some manufacturers of fluoroscopic equipment store the $K_{a,r}$ resulting from each digital acquisition series — and in some cases stored fluoroscopy loops — in private tags within the DICOM header. Public tags can be accessed on many PACS (Fig. 1(a)) or by using image analysis software such as ImageJ (National Institutes of Health, Bethesda, MD) (Fig. 1(b)). Private tags are often inaccessible using PACS or ImageJ but can be accessed with software such as MATLAB (MathWorks, Natick, MA) (Fig. 1(c)).

In some cases, the DICOM header can be mined to gather most, if not all, of the information contained within a proprietary dose report or DICOM Radiation Dose Structured Report. In all cases, the DICOM header can be mined to gather supplemental information that is useful for converting $K_{a,r}$ to PSD.

#### A.2 Using dose reports

**Figure 2:**
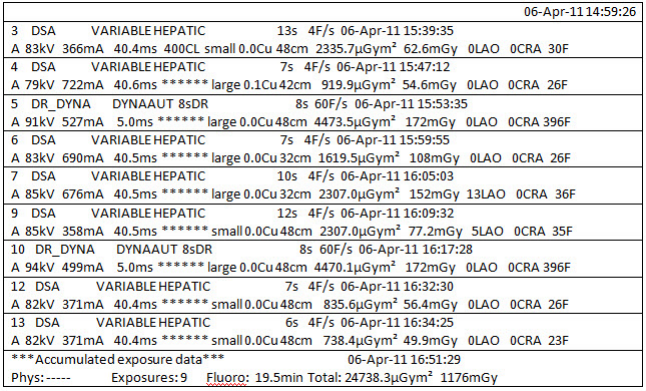
Format of a proprietary dose report.

Some manufacturers of fluoroscopic equipment generate a dose report at the end of a procedure, with separate instances created for each digital acquisition series and totals for certain dose metrics. This report may contain a variety of information including technical factors, $K_{a,r}$, KAP, and fluoroscopy time (Fig. 2). Such reports provide a convenient starting point for PSD calculation, as most of the necessary information is contained within the report. The newest versions of equipment from many vendors now also support the DICOM Radiation Dose Structured Report.^(^ ^6^ ^)^

#### A.3 Wholesale versus piecemeal calculation

Calculating the contribution to $K_{a,r}(d)$ for each individual image or series can be time‐consuming. If the same FOV and a similar X‐ray field size (i.e., beam collimation) were used throughout the procedure, the use of a single X‐ray field area for wholesale calculation of $K_{a,r}(d)$ is likely a safe simplification. If the FOV or X‐ray field size changed frequently throughout the procedure, however, a piecemeal calculation of $K_{a,r}(d)$ is the more accurate approach.

### B. Calculation of peak entrance skin air kerma

After the $K_{a,r}(d)$ has been determined, the next step in estimating the PSD is calculation of the peak ESAK. This step involves an inverse square law correction and a correction for attenuation by the table pad and table.

#### B.1 Inverse square law correction

While the IRP was defined to correspond approximately with the entrance skin surface of a patient in interventional cardiology, it is often the case that outside of interventional cardiology the IRP does not correspond with the entrance skin surface of the patient. In fact, it should not correspond with the patient surface if good practice is used in interventional radiology (i.e., the patient is moved as far as possible from the X‐ray source).

One method for determining the location of the patient with respect to the IRP is the use of information from the DICOM header (Table 2), in particular tag (0018,0111), “Distance Source to Patient”. The DICOM conformance statement provided by the manufacturer of a fluoroscope can be consulted to determine the actual location specified by this tag. We have noticed that this tag may differ from the DICOM standard, in some cases recording the source‐to‐table distance, which is a good approximation of the source‐to‐patient distance. Measurements can be performed to determine what distance is being stored in the tag. If the source‐to‐table distance is not available in the DICOM header, it must be measured and recorded manually. This is absolutely necessary, as the height of the physician strongly influences the table height — and therefore the SPD — during an FGI. Technologists can be instructed to measure the source‐to‐table distance for all cases, for known high dose FGI, or at a certain trigger level based on fluoroscopy time or $K_{a,r}$.

After determining the SPD, the following calculation can be performed:

(3) $$K_{a,table}=K_{a,r}\times {\left(\frac{d_{source-to-IRP}}{d_{source-to-patient}}\right)}^{2}$$

where $K_{a,table}$ is the air kerma at the surface of the table.

#### B.2 Correction for attenuation of table pad and table

While the table attenuates the primary beam by a small amount, the use of a thick table pad constructed of viscoelastic polyurethane foam can result in a substantial reduction in the entrance air kerma rate.^(^ ^7^ ^)^ If a machine‐reported KAP or $K_{a,r}$ value is used, it is important to understand how the KAP or $K_{a,r}$ is calibrated by the manufacturer, specifically with or without the table and/or pad in the X‐ray beam, as this will determine whether the correction factor for this step is calculated with or without the table and/or pad in the beam.

Correction factors for the table and table pad should be based on broad‐beam attenuation and determined using an X‐ray spectrum as similar as possible to that used during the procedure under investigation. If the $K_{a,r}$ (d) is measured (Scenario 2), the table pad and table can be left in the beam and the correction included in the measurement.

Upon determining the correction factor for the table pad and/or table (t), the following correction can be made:

(4) $$ESAK=K_{a,table}\times t$$

Certain circumstances, such as lateral projections or biplane imaging, may result in situations in which the table is not in the X‐ray beam. In this case, $K_{a,\text{table}}$ in Eq. (3) would be replaced by ESAK, and the correction described in Eq. (4) would be unnecessary.

### C. Calculation of peak skin dose

The final step in the reconstruction process is calculation of the PSD. The PSD can be calculated from the peak ESAK by applying the BSF and the f‐factor.

#### C.1 The backscatter factor

**Figure 3:**
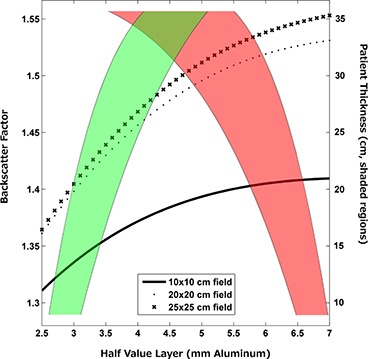
Backscatter factor as a function of patient size for a range of beam qualities. Red shading indicates typical values for fluoroscopy, green shading indicates typical values for digital acquisition imaging. To find the BSF for a given patient size and imaging mode: 1. Follow the value for the patient size across to the appropriate shaded region; 2. Follow the shaded region to the Half Value Layer axis; 3. Draw a line from the intersection of the shaded region with the Half Value Layer axis to the X‐ray field size used; 4. Draw a line from the X‐ray field size curve to the BSF axis to find the BSF. Data used to generate figure were taken from ICRU.^(^ ^8^ ^)^

At diagnostic X‐ray energies, many X‐rays are backscattered at their first interaction in tissue and therefore can undergo multiple interactions contributing to skin dose. The BSF is the ratio of the air kerma measured with a patient or phantom in the X‐ray beam to the air kerma measured at the same location without a patient or phantom in the X‐ray beam. Tables of BSF determined both experimentally^(^ ^8^ ^)^ and through simulation^(^ ^9^ ^)^ as a function of beam quality, substance, and X‐ray field size have been published. We have used the data from Petoussi‐Henss et al.^(^ ^9^ ^)^ to assemble a graphic plotting the BSF as a function of patient size for a range of beam qualities (Fig. 3). Accurate determination of the BSF requires that the beam quality, tissue, and X‐ray field size be known. Methods for determining the X‐ray field size were discussed earlier in the context of converting KAP values to $K_{a,r}$ values. Alternatively, an “effective” X‐ray field size for the entire study can be derived by dividing the KAP by the $K_{a,r}$, if both quantities are available. The substance of interest in the case of skin injuries is, of course, soft tissue. Beam quality in fluoroscopic imaging is a function of kV, inherent and added filtration in the collimator, and any removable filtration added to harden the X‐ray beam. Modern fluoroscopes often include small thicknesses of aluminum and/or small thicknesses of copper to increase beam quality and reduce skin dose. The DICOM header can again be mined for data pertaining to beam quality, including the kV and in some cases the type and amount of any added filtration (Table 2). Table pads can also affect the beam quality.^(^ ^7^ ^)^ The significance of the effect of the table pad on beam quality depends on the presence of other removable filtration in the X‐ray beam. If additional copper filtration is used, the effect of the table pad is likely to be small. If no other filtration is present, the effect of the table pad may be significant. It is suggested that beam quality be represented in terms of the half‐value layer (HVL) instead of attempting to exactly match the kV and filtration. One must also consider the fact that the BSF can vary from series to series if the beam quality or X‐ray field size changes.

#### C.2 The f‐factor

Different tissues absorb ionizing radiation more or less efficiently depending on both the tissue type and X‐ray beam quality. Therefore, a beam of ionizing radiation will deposit more of its energy in certain tissue types than others. The f‐factor is calculated as:

(5) $$f_{medium}={\left(\frac{\mu_{en}}{\rho }\right)}_{medium}/{\left(\frac{\mu_{en}}{\rho }\right)}_{air}$$

and has been tabulated for several tissue types.^(^ ^10^ ^)^ (Technically speaking, the f‐factor converts exposure to dose in a medium. However, the term “f‐factor” is commonly used to refer to a similar factor used to convert air kerma to dose in a medium). Tables of f‐factors are provided in this manuscript for convenience. Table 3 lists the f‐factor as a function of beam quality (HVL), while Table 4 lists the f‐factor as a function of kVp for both fluoroscopic and digital acquisition modes, for cases where the beam quality is not explicitly known. The f‐factors in both tables are for ICRU soft tissue.^(^ ^8^ ^)^

**Table 3:** Values of the f‐factor for ICRU soft tissue for beam qualities commonly encountered in fluoroscopy.

*HVL (mm Al)*	*f‐factor*
3.0 – 3.5	1.058
3.5 – 4.0	1.059
4.0 – 4.5	1.061
4.5 – 5.0	1.062
5.0 – 5.5	1.062
5.5 – 6.0	1.063
6.0 – 6.5	1.066
6.5 – 7.0	1.068

**Table 4:** Values of the f‐factor for ICRU soft tissue as a function of kVp, for cases where the HVL is not known.

	*f‐factor*	
*kVp*	*Fluoroscopic Mode*	*Digital Acquisition Mode*
60	1.061	1.056
65	1.063	1.058
70	1.065	1.059
75	1.066	1.061
85	1.068	1.063
95	1.069	1.066

Upon determining the BSF and f‐factor, the PSD can be calculated as

(6) $$PSD=ESAK\times BSF\left(HVL,A_{skin},tissue\right)\times f_{tissue}(HVL)$$

**Figure 4:**
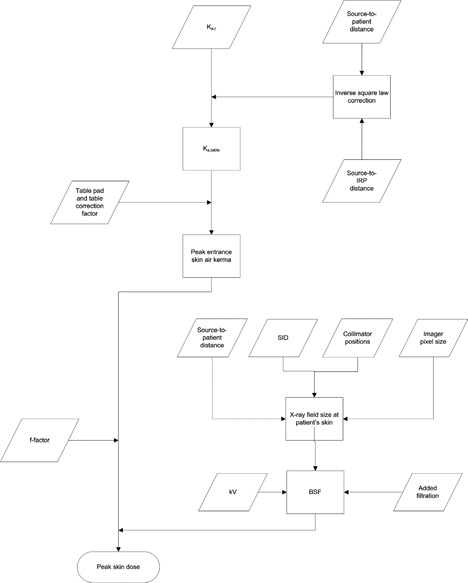
Flowchart outlining the basic steps in calculating the PSD from $K_{a,r}$. A similar workflow would be used to calculate the PSD from the KAP, with the added step of converting from KAP to $K_{a,r}$ by dividing by the X‐ray field size.

The aforementioned steps are summarized in Fig. 4.

## III. ESTIMATING THE CONTRIBUTION OF FLUOROSCOPY TO THE PEAK SKIN DOSE

The contribution of fluoroscopic imaging to the PSD can vary widely, ranging from almost 100% if little or no digital acquisition imaging is used, to as low as 10%–20% if many DAS are acquired. Although digital acquisition imaging is often the major contributor to PSD, it is never safe to assume that the contribution of fluoroscopy is negligible. However, estimating the contribution of fluoroscopy to the PSD can be a challenging task, as images are rarely available and dose metrics may not be available.

**Method 1: Wholesale calculation of peak skin dose**

With this method, the contribution of fluoroscopy to the PSD is not explicitly considered. Instead, single values of the BSF, f‐factor, inverse square correction, and table pad correction representative of those encountered in the course of the procedure are used to convert the total reference point air kerma, $K_{a,r}(t)$, to PSD. The $K_{a,r}(t)$ value includes contributions from both fluoroscopy $\left[K_{a,r}(f)\right]$ and digital acquisition imaging $\left[K_{a,r}(d)\right]$:

(7) $$K_{a,r}(t)=K_{a,r}(f)+K_{a,r}(d)$$

and therefore no separate consideration needs to be made for fluoroscopy. This method may reduce the accuracy of the estimation of the contribution of digital acquisition imaging to PSD, as individual DAS are no longer considered separately.

**Method 2: Separation of fluoroscopic air kerma from total air kerma**

If a dose report is available, or if $K_{a,r}(d)$ values are available in the DICOM header of DAS and $K_{a,r}(t)$ is known, $K_{a,r}(f)$ can be considered separately from $K_{a,r}(t)$. The sum of the $K_{a,r}(d)$ contribution of all DAS can be subtracted from the reported $K_{a,r}(t)$ to determine $K_{a,r}(f)$. Representative values for the BSF, f‐factor, inverse square, and table pad corrections can be chosen to calculate the contribution of fluoroscopy to the PSD using $K_{a,r}(f)$ and the steps outlined in Section II above. These factors can be derived in a number of ways. They can be estimated based on patient thickness and measured data, estimated from a look‐up table relating acquisition technical factors to fluoroscopic technical factors, mean or median factors from the DAS, or estimated from the DICOM header of stored fluoroscopy loops. This latter method may result in a more accurate estimate of PSD at the expense of additional calculation time.

**Method 3: Measurement of fluoroscopic air kerma rate**

If no dose report is available and $K_{a,r}(t)$ is not available, the fluoroscopic air kerma rate can be measured. The staff should be interviewed to determine the mode of operation of the system and the approximate table height. The patient dimension in the direction of the X‐ray beam should be determined from either cross‐sectional images or data contained in the medical record. The fluoroscopic air kerma rate measured under conditions approximating those used during the procedure can then by multiplied by the recorded fluoroscopy time to estimate $K_{a,r}(f)$, and Method 2 can be used from this point to calculate the contribution of fluoroscopy to PSD. If the fluoroscopy time is unavailable, an accurate estimate of the contribution of fluoroscopy to $K_{a,r}(t)$ cannot be made.

## IV. CALCULATING THE TOTAL PEAK SKIN DOSE

The total PSD is the sum of the contributions of digital acquisition imaging and fluoroscopy to PSD.

## V. SOURCES OF ERROR

### A. Distribution of dose over multiple skin sites

A common misconception is that dose can be “spread” over the skin by rotating the C‐arm during a procedure, thereby reducing the PSD. Although this strategy can be effective in interventional cardiology,^(^ ^11^ ^,^ ^12^ ^)^ it has recently been shown that this is not the case for interventional radiology.^(^ ^13^ ^)^ Therefore, we recommend that for procedures using modest C‐arm rotation angles, all $K_{a,r}$ be attributed to a single skin site. There are circumstances in which skin dose can safely be considered to be distributed amongst two distinct skin sites, such as biplane laboratories used for neuroradiology or cardiology, and for procedures performed in a “biplanar” fashion with a single‐plane unit (e.g., a vertebroplasty procedure in which alternating views are acquired with PA and lateral projections).

### B. Rotational angiography

Manufacturers of fluoroscopic equipment now offer rotational angiography on C‐arm fluoroscopes. The images acquired during a rotational angiography run are most often used to reconstruct CT‐like images. As the C‐arm rotates through a large angular range, between 210° and 360°, the radiation dose is distributed across a large area of the patient's skin. One may wish to apply an empirically determined adjustment to the calculated PSD if multiple rotational angiography runs were performed in the course of a study, as the peak ESAK may be substantially less than would be indicated by the $K_{a,r}$. The concept of the dose index,^(^ ^14^ ^)^ defined as the ratio of the PSD to the $K_{a,r}$, may be useful in these cases.

### C. Accuracy of reported dose metrics

The estimated PSD is only as accurate as the dose metrics reported by the fluoroscopic equipment. The reported dose metrics are required by international standards^(^ ^3^ ^)^ and United States law^(^ ^4^ ^)^ to meet certain minimum requirements for accuracy. However, these minimum requirements (±35%) can lead to large uncertainties in estimated PSD values. We recommend that the accuracy of reported dose metrics be evaluated on an annual basis and recalibration performed when deemed necessary by a qualified medical physicist. The amount of error that is acceptable can be estimated based on the contribution of other factors to the total error, including the calculation methodology used. The qualified medical physicist should also consider that the accuracy of KAP meters is influenced by kV and that the accuracy of calculated $K_{a,r}$ values is influenced by errors in the X‐ray field size. Some manufacturers of fluoroscopic equipment use lookup tables to estimate KAP and $K_{a,r}$ using technical factors such as kVp, mA, and added filtration. The accuracy of these estimates should be evaluated.

The data from the DICOM header should be verified with measurements. It has been our experience that some data found in the DICOM header of certain manufacturers do not reflect the DICOM standard (e.g., actual table position instead of isocenter position being stored in the “Distance Source to Patient” tag). Also, we have found that the units for quantities stated in some private tags are incorrect (e.g., a DICOM conformance statement may list units of “mGy” for dose when, in fact, the units are “$\mu \text{Gy}\times 10^{1}$”).

### D. Putting errors in perspective

The error in the calculated PSD will also be influenced by procedural factors such as rotation of the C‐arm and use of rotational angiography. It is not hard to imagine that errors as low as 10% may be achievable with an accurately‐calibrated KAP meter for a procedure that uses only a PA projection with no rotational angiography and for which a dose report is available, when digital acquisition series are considered piecemeal and fluoroscopy is considered separately. Larger errors owing to simplification would be expected if the PSD is calculated in a wholesale fashion, but this method would still be expected to yield more accurate results than attempting to reconstruct a procedure by measuring digital acquisition and fluoroscopic air kerma rates. Reconstructing a procedure with staff interviews, phantoms, and measurements may result in large errors. Finally, in some cases, estimating the PSD may be impossible if no data or images are available for review.

We must consider the goal of estimating PSD — knowledge of the PSD is a tool for appropriate medical management of the patient after a high‐dose FGI. The most recent report on radiation‐induced skin changes groups the changes into dose “bands” based on the PSD.^(^ ^15^ ^)^ These bands are 0–2 Gy, 2–5 Gy, 5–10 Gy, 10–15 Gy, and greater than 15 Gy. Therefore, it is most important to accurately determine into which band a patient falls rather than to estimate the PSD with an accuracy of, for example, ±10%. Viewed in this light, we can see that some simplification, for example, “wholesale” consideration of $K_{a,r}(t)$, is in most cases likely to be sufficient for estimation of the PSD.

## VI. SUMMARY

In the absence of physical measurements, the PSD experienced by a patient undergoing an FGI can be estimated using dose metrics and other data that are commonly available on modern fluoroscopes. By realizing what data are needed to estimate the PSD, the medical physicist can plan ahead and implement processes to collect necessary data that are not available on the imaging system or to collect supplementary data to aid in the calculation process. Also, by understanding the benefits and limitations of different methods for dose estimation, the medical physicist can offer a realistic assessment of the potential accuracy of an estimate of the PSD.

An important area of future work is the physical measurement of PSD for a variety of FGI, and comparison of the measurement results to calculations performed using these methods with standardized dose metrics. The algorithms described in this work can easily be implemented in a programming language such as MATLAB or even in a simple spreadsheet. Information from the DICOM Radiation Dose Structured Report, a proprietary dose report, or simple machine‐reported values of $K_{a,r}$ or KAP can be parsed and fed into the algorithm along with data measured by a medical physicist, resulting in rapid estimations of peak skin dose without the inconvenience and expense of making physical measurements. However, the accuracy of these techniques must first be evaluated by comparison with physical measurements.

## ACKNOWLEDGMENTS

The authors would like to thank Michael Worley for his editorial assistance.

## References

[1] Balter S . Methods for measuring fluoroscopic skin dose. Pediatr Radiol. 2006;36(Suppl 2):136–40. 16862416 10.1007/s00247-006-0193-3 PMC2663643

[2] Miller DL , Balter S , Cole PE , et al. Radiation doses in interventional radiology procedures: The RAD‐IR study: Part II: skin dose. J Vasc Interv Radiol. 2003;14(8):977–90. 12902555 10.1097/01.rvi.0000084601.43811.cb

[3] International Electrotechnical Commission . Medical electrical equipment ‐ Part 2–43: Particular requirements for the safety of X‐ray equipment for interventional procedures. IEC 60601‐2‐43. Geneva: IEC; 2000.

[4] United States Food and Drug Administration . Code of Federal Regulations, 21CFR1020.32(XX), Part 1020 ‐ Performance standards for ionizing radiation emitting products. Silver Springs, MD: FDA; 2002.

[5] National Electrical Manufacturer's Association . Digital imaging and communications in medicine (DICOM) Part 3: information object definitions (PS 3.3‐2009). Rosslyn (VA): NEMA; 2009 p. 1286 Available at ftp://medical.nema.org/medical/dicom/2009/09_03pu3.pdf; accessed 4/201.

[6] National Electrical Manufacturer's Association . Digital imaging and communications in medicine (DICOM) Supplement 94:diagnostic X‐ray radiation dose reporting (Dose SR). Rosslyn (VA): NEMA; 2005 P. 36 Available at ftp://medical.nema.org/medical/dicom/final/sup94_ft.pdf; accessed 4/2011.

[7] Geiser WR , Huda W , Gkanatsios NA . Effect of patient support pads on image quality and dose in fluoroscopy. Med Phys. 1997;24(3):377–82. 9089590 10.1118/1.597906

[8] ICRU . Tissue substitutes in radiation dosimetry and measurement. ICRU Report 44. Bethesda, MD: ICRU Publications; 1989.

[9] Petoussi‐Henss N , Zankl M , Drexler G , Panzer W , Regulla D . Calculation of backscatter factors for diagnostic radiology using Monte Carlo methods. Phys Med Biol. 1998;43(8):2237–50. 9725601 10.1088/0031-9155/43/8/017

[10] Johns HE and Cunningham JR, eds. The physics of radiology. 4th ed. Springfield, IL: Charles C Thomas; 1983 p. 796.

[11] Kuon E , Dahm JB , Empen K , Robinson DM , Reuter G , Wucherer M . Identification of less‐irradiating tube angulations in invasive cardiology. J Am Coll Cardiol. 2004;44(7):1420–28. 15464322 10.1016/j.jacc.2004.06.057

[12] Kuon E , Glaser C , Dahm JB . Effective techniques for reduction of radiation dosage to patients undergoing invasive cardiac procedures. Br J Radiol. 2003;76(906):406–13. 12814927 10.1259/bjr/82051842

[13] Pasciak AS and Jones AK . Does “spreading” skin dose by rotating the C‐arm during an intervention work? J Vasc Interv Radiol. 2011;22(4):443–52. 21367618 10.1016/j.jvir.2010.12.025

[14] Miller DL , Balter S , Noonan PT , Georgia JD . Minimizing radiation‐induced skin injury in interventional radiology procedures. Radiology. 2002;225(2):329–36. 12409563 10.1148/radiol.2252011414

[15] Balter S , Hopewell JW , Miller DL , Wagner LK , Zelefsky MJ . Fluoroscopically guided interventional procedures: a review of radiation effects on patients' skin and hair. Radiology. 2010;254(2):326–41. 20093507 10.1148/radiol.2542082312

