# Erratum: Calculating the peak skin dose resulting from fluoroscopically guided interventions. Part I: Methods

**DOI:** 10.1120/jacmp.v15i4.4986

**Published:** 2014-07-08

**Authors:** A. Kyle Jones, Alexander S. Pasciak

**Affiliations:** ^1^ Department of Imaging Physics Division of Diagnostic Imaging, The University of Texas M. D. Anderson Cancer Center Houston TX 77030; ^2^ Department of Radiology University of Tennessee Medical Center Knoxville TN 37920

It has come to our attention that there is a mistake with Eq. (1) in our article: *Calculating the peak skin dose resulting from fluoroscopically guided interventions. Part I: Methods*.^(1)^ Equation (1) in this article should read:

Askin=[(|CLef t−CRight|×(p10))×(|CUpper−CLower|×(p10))]×(SPDSID)2

The squared term was missing from the factor $\left(\frac{SPD}{SID}\right)$ in the original article. This term needs to be squared, as it applies to both the horizontal and vertical collimator dimensions. The correct form of the expression was used in the examples in the companion article: *Calculating the peak skin dose resulting from fluoroscopically guided interventions. Part II: Case studies*.^(2)^ We wish to thank Guang Li, PhD, for bringing this omission to our attention.

We would like to take this opportunity to make one additional clarification regarding these articles. The DICOM tag 0x00181111 (Distance Source to Patient) must be used carefully when calculating peak skin dose. The actual distance stored in this tag is affected by the choice of method used to calibrate distances in the image. In many cases, the distance stored in this tag is calculated based on the default table‐to‐object (TOD) distance used in the configuration files of the fluoroscope. However, the TOD setting can be changed by the operator or another method may be used to calibrate the images (e.g., catheter choice, an object such as a coin placed in the field of view, etc.). It is important that this be considered when using the distance stored in this tag.

## Supporting information

Supplementary Material
