# Supplementary material for: Erratum: Calculating the peak skin dose resulting from fluoroscopically guided interventions. Part I: Methods
Source: J Appl Clin Med Phys. 2014 Jul 8;15(4):402. doi: 10.1120/jacmp.v15i4.4986 (PMC6458842; doi:10.1120/jacmp.v15i4.4986)
Supplement: Supplementary file 1 — Supplementary Material [file ACM2-15-402-s001.doc]

Erratum: “Calculating the peak skin dose resulting from fluoroscopically guided interventions. Part I: Methods”

A. Kyle Jones

Department of Imaging Physics, Division of Diagnostic Imaging

MD Anderson Cancer Center

*1400 Pressler, Unit 1432, Houston, TX 77030*

[kyle.jones@mdanderson.org](mailto:kyle.jones@mdanderson.org)

Alexander S. Pasciak

Department of Radiology

University of Tennessee Medical Center, Knoxville

*1924 Alcoa Highway, Knoxville, TN 37920*

[APasciak@mc.utmck.edu](mailto:APasciak@mc.utmck.edu)

Corresponding author: A. Kyle Jones

[kyle.jones@mdanderson.org](mailto:kyle.jones@mdanderson.org)
